# The *Hv-SGT1* Gene from *Haynaldia* *villosa* Contributes to Resistances Towards Both Biotrophic and Hemi-Biotrophic Pathogens in Common Wheat (*Triticum aestivum* L.)

**DOI:** 10.1371/journal.pone.0072571

**Published:** 2013-09-03

**Authors:** Liping Xing, Chen Qian, Aizhong Cao, Yingbo Li, Zhengning Jiang, Minghao Li, Xiahong Jin, Jiameng Hu, Yiping Zhang, Xiue Wang, Peidu Chen

**Affiliations:** The National Key Laboratory of Crop Genetics and Germplasm Enhancement, Nanjing Agricultural University, Nanjing, China; University of Geneva, Switzerland; Cankiri Karatekin University, Turkey

## Abstract

The SGT1 protein is essential for R protein-mediated and PAMPs-triggered resistance in many plant species. Here we reported the isolation and characterization of the *Hv-SGT1* gene from *Haynaldia* *villosa* (2n = 14, VV). Analysis of the subcellular location of Hv-SGT1 by transient expression of a fusion to GFP indicated its presence in the cytoplasm and nucleus. Levels of *Hv-SGT1* transcripts were increased by inoculation with either the biotrophic pathogen *Blumeria* *graminis* DC. f. Sp. *tritici* (*Bgt*) or the hemi-biotrophic pathogen *Fusarium* *graminearum* (*Fg*). Levels of *Hv-SGT1* showed substantial increase following treatment with H_2_O_2_ and methyl jasmonate (MeJA), only slightly induced following exposure to ethephon or abscisic acid, but not changed following exposure to salicylic acid. The demonstration that silencing of *Hv-SGT1* substantially reduced resistance to *Bgt* indicated that Hv-SGT1 was an essential component of disease resistance in *H* *. villosa* . The over-expression of *Hv-SGT1* in Yangmai 158 enhanced resistance to powdery mildew, and this correlated with increased levels of whole-cell reactive oxygen intermediates at the sites of penetration by the pathogens. Compared with wild-type plants, the expression levels of genes related to the H_2_O_2_ and JA signaling pathways were lower in the *Hv-SGT1* silenced plants and higher in the *Hv-SGT1* over-expressing plants. Therefore, the involvement of *Hv-SGT1* in H_2_O_2_ production correlates with the hypersensitive response and jasmonic acid signaling. Our novel demonstration that wheat with over-expressed *Hv-SGT1* showed enhanced resistance to both powdery mildew and FHB suggests that it could served as a transgenic genetic resource in wheat breeding for multiple disease resistance.

## Introduction

Plants activate various regulated defense mechanisms in response to a broad range of pathogen invasions. Pathogen-associated molecular patterns (PAMPs)-triggered immunity (PTI) and effector-triggered immunity (ETI) are the two main classes of plant innate immune responses [1,2]. Several recent studies have indicated that plant SGT1 (suppressor of the G2 allele of skp1) was a critical signaling component required for both PTI and ETI mediated host cell death in several plant species against various plant pathogens, including fungi, bacteria and viruses [3–8].

The SGT1 protein was first identified as a suppressor of *skp1–4*, which was involved in kinetochore assembly in yeast [9]. Hereafter, conserved SGT1 homologues were found in many eukaryotes, and were shown to participate in various aspects of plant biology, including plant defense responses and development. All SGT1 proteins had three distinct regions: a tetratricopeptide repeat (TPR) domain, a CS (present in CHP and SGT1 proteins) domain, and an SGS (SGT1-specific sequence) domain. The CS domain interacted with different protein complexes to affect a range of biological processes [10].

In plants, SGT1 positively regulates many plant disease-resistance (R)-gene-mediated race-specific disease resistances [5,11]. Mutation or silencing of *SGT1* will compromised disease resistance mediated by plant nucleotide-binding domain and leucine-rich repeat-containing (NLR) type *R* genes, such as *MLA*, *N*, *Bs2*, *Bs4*, *Rx*, *RPS4*, *Prf*, *Mi*, *I2*, *R3a*, and *Lr21* or by non-NLR-type sensors such as Cf 4,9, or *RPW8* [12]. Over-expression of *SGT1* will sometimes enhance plant disease resistance. Over-expression of *NbSGT1* in *Nicotiana* *benthamiana* accelerated the development of hypersensitive response (HR) during *R-*gene-mediated disease resistance [8]. Over-expression of *OsSGT1* in rice significantly increased basal resistance to a virulent bacterial blight *Xanthomonas* *oryzae* pv. *oryzae* *PXO99* and four virulent blast fungal *Magnaporthe* *oryzae* races [13]. Expression of SGT1 was tightly related to *R*-gene expression, the HR, and the activation of some R proteins [14,15]. Additionally, *SGT1* is also required in some cases of non-host plant resistance [16].

During disease resistance, SGT1 appears to interact with molecular chaperones (RAR1, HSP70 or HSP90) to mediate target protein binding to initiate a specific signaling cascade that confers resistance. For example, the SGT1-RAR1-HSP90 complex was essential for *Lr21*-triggered wheat disease resistance to leaf rust [17]. The SGT1 protein interacted physically with SKP1, a part of the SCF E3 ubiquitin ligase, suggesting that its functions in the proteasome-mediated protein degradation pathway [16]. Both the *NbSGT1* and *NbSKP1* genes played important roles in the *N*-mediated resistance response to TMV [6].

Fungal pathogens can be classified as biotrophs, necrotrophs and hemi-biotrophs. Two globally important diseases that affect common wheat, powdery mildew and Fusarium head blight (FHB) (or scab), are caused by the biotrophic *Blumeria* *graminis* DC. f. sp. *tritici* (*Bgt*) and the hemi-biotrophic *Fusarium* *graminearum* (*Fg*) respectively. However, the mechanisms responsible for the resistance to biotrophs and necrotrophic pathogens are quite different. Active cell death (also known as the HR) in host-pathogen interaction sites is an effective resistance approach adopted by plant hosts to restrict the development of biotrophs. However, virulent necrotrophic pathogens must be able to damage or kill host tissues (necrosis) to support their successful infection and survival. Accordingly, it was proposed that it is difficult to co-regulate resistances to both the biotrophic and necrotrophic pathogens. Recent studies showed that SGT1 promotes both the HR and resistance to biotrophic pathogens, while suppressing resistance to necrotrophic pathogens. Silencing of *SGT1* expression reduced necrosis, which in turn compromised resistance to the barley biothoph *Blumeria* *graminis* f. sp. *Hordei* (*Bgh*) [18] and the wheat biotroph *Pucciniastriiformis* f. sp. *tritici* (*Pst*) [17], while enhancing the resistance of tobacco to the necrotrophic pathogen *Botrytis cinerea* [19]. The resistance mechanisms used by hemi-biotrophs, were even more complicated. Cuzick et al. [20] reported that a lack of *SGT1b* reduced host-cell death and promoted the pathogenesis of the hemi-biotrophic fungal pathogen *Fusarium* *culmorum* . However, the potential role of *SGT1* in powdery mildew resistance and FHB resistance in wheat was not known yet.

*Haynaldia* *villosa* , a diploid wild member of the genus *Triticeae* of the *Poaceae*, displays substantial broad-spectrum resistance to *Bgt*, and the disease resistance gene *Pm21* that is responsible for this resistance has been transferred to common wheat by chromosome engineering [21]. Microarray analysis conducted in our laboratory on *H* *. villosa* plants that were either inoculated with *Bgt* or treated as controls were used to identify key genes in the response network [22] that mediated resistance to *Bgt* resistance. This included a key member of the *Pm21* locus, the *Stpk-V* gene [23]. Besides *Stpk-V*, homologues of *SGT1*, *RAR1*, and *HSP90* were also found to be induced in *Bgt* inoculated *H* *. villosa* . Here, we focused on the function of *SGT1* in mediating the resistance of wheat towards fungal pathogens. A full-length *H* *. villosa* *SGT1* gene was cloned and characterized in detail. Over-expression of *Hv-SGT1* in common wheat and silencing of *Hv-SGT1* in *H* *. villosa* revealed its pivotal roles in resistance to both the biotrophic pathogen *Bgt* and hemi-biotrophic pathogen *Fg*. It was shown that the different responses to attacks by these different pathogens are associated with activation or depression of the H_2_O_2_- and JA-mediated defense pathways.

## Results

### Identification of the *Hv-SGT1*

The Genechip microarray data collected from *H* *. villosa* (Cao et al. 2006a) plants inoculated with *Bgt* enabled us to design degenerate primer pairs to clone a full-length cDNA of *Hv-SGT1* from *H* *. villosa* using RT-PCR and RACE (rapid-amplification of cDNA ends). The 1,429-bp cDNA fragment, which included the complete 1,122-bp open reading frame (ORF) that encoded a protein of 376 amino acid residues, was assigned an Accession No. JX885369 in GenBank.

**Figure 1:**
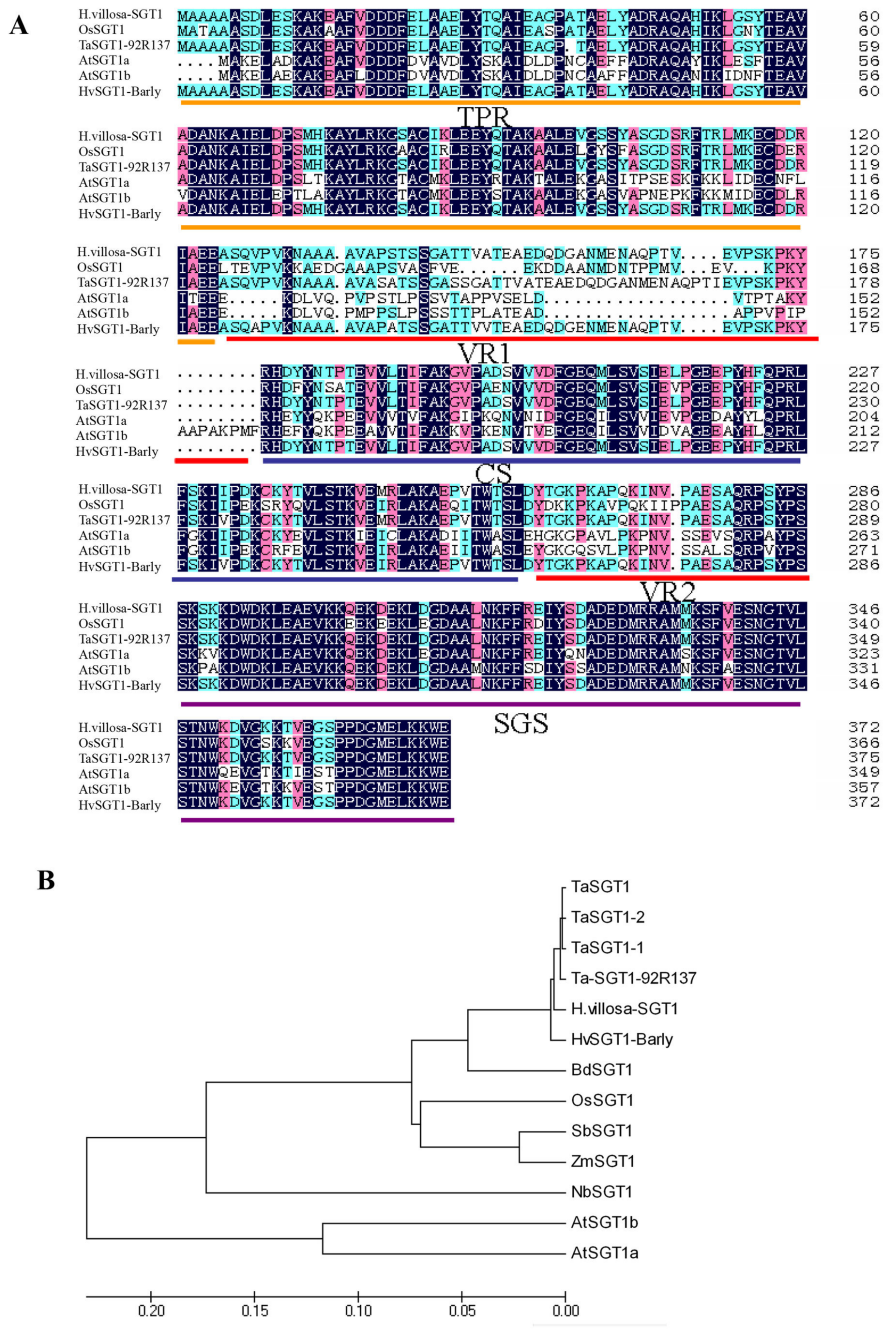
Analysis of primary structure and conserved domains of SGT1 proteins. (A) Sequence alignment analysis and the predicted conserved domains of SGT1 proteins. GenBank accession numbers: Rice (AAF18438), Wheat (EF546432.1), *Arabidopsis* (AF439975, AF439976), Barley (AF439974). The black (100%), pink (80%), and blue (60%) boxes represent levels of amino acid identity or similarity. The conserved domains were underlined. (B) The phylogenetic analysis of the amino acid sequence of *SGT1* genes from different species. GenBank accession numbers: OsSGT1 (AAF18438), TaSGT1 (ABQ23992.1), TaSGT1-1 (ABO18602.1), TaSGT1-2 (ABO18603.1), HHvSGT1-Barley (AF439974), BdSGT1 (XP_003569394.1), SbSGT1 (EES01101.1), Zm-SGT1 (ACG34278.1), NbSGT1 (AAW82048.1), AtSGT1a (AF439975) and AtSGT1b (AF439976). The tree was generated by ClustalX1.83 analysis with the corrected full-length Hv-SGT1 protein sequences using Neighbor-Joining method (MEGA4.0 software). The bar beneath the dendrogram represents a distance of 0.05 change per amino acid.

The predicted Hv-SGT1 protein contained five domains: a tetratricopeptide repeat domain (TPR), a P23 region with two variable domains (VR1 and VR2), a CS motif (present in the metazoan CHORD and SGT1 proteins), and an SGS (SGT1-specific) motif. Sequence alignment of SGT1 proteins from different species showed that Hv-SGT1 had the conserved domains present in other SGT1 proteins (Figure 1A). Phylogenetic analysis showed that Hv-SGT1 displayed different degree of similarity to other plant SGT1s, with the highest homology (98%) to barley SGT1. The SGT1 cloned from the common wheat line 92R137 (designated as Ta-SGT1-92R137) shared 95% s amino acid similarity with Hv-SGT1 (Figure 1B).

**Figure 2:**
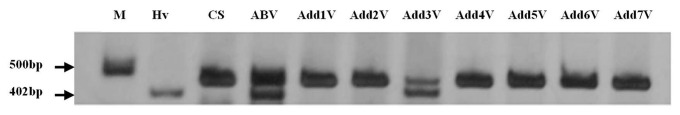
Chromosomal location of *Hv-SGT1* determined using the wheat–*H. villosa* addition lines as the templates. The arrow shows the specific amplicon from *H* *. villosa* .. M: DNA marker DL2000. HV:H*. villosa*, CS: wheat *cv*. Chinese spring, ABV: *T* *. durum* *-* *H* *. villosa* amphiploid, 2n=42, genome AABBVV, Add1V to Add7V: *T. aestivum-* *H* *. villosa* addition line, each contains one pair of chromosomes of *H* *. villosa* from 1V to 7V in the common wheat background, 2n=44, genome AABBDD add 1V1V~7V7V.

The chromosomal location of *Hv-SGT1* was determined following PCR-based amplification using DNA from the wheat- *H* *. villosa* addition lines as template. A 402-bp product was found in *H* *. villosa* , a *T* *. durum* – *H* *. villosa* amphiploid, and a wheat- *H* *. villosa* addition line DA3V, but not in Chinese spring and the remaining addition lines tested. This indicated that *Hv-SGT1* was located on chromosome 3V (Figure 2).

**Figure 3:**
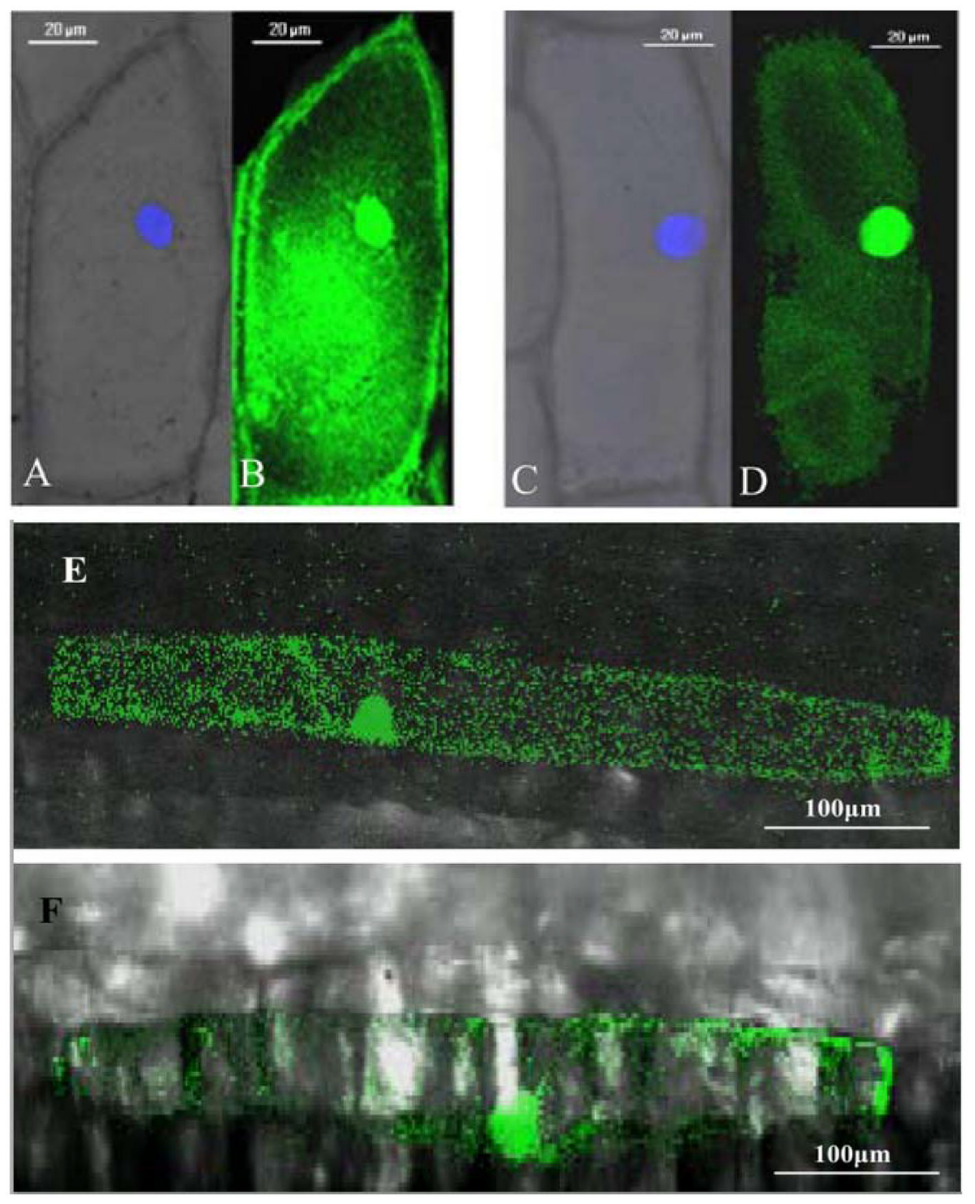
Subcellular localization of Hv-SGT1-GFP fusion protein by transient expression via biolistic bombardment. (A–B) Detected GFP under blue emitting light (nucleus stainning with DAPI) and visible light (merged), and green emitting light of the onion epidermis cell and (E) in the merged figure of the *H* *. villosa* epidermis cell, indicating that GFP was sub-celluarly located to the whole cell. (C–D) Detected Hv-SGT1-GFP under blue emitting light (nucleus stainning with DAPI) and visible light (merged), and green emitting light of the onion epidermis cell and (F) in the merged figure of the *H* *. villosa* epidermis cell, indicating that the Hv-SGT1 was subcelluarly located to the nuclei and cytoplasm.

A construct that placed an in-frame fusion of the *Hv-SGT1* coding sequence with the coding region for green fluorescent protein (Hv-SGT1::GFP) under the control of the cauliflower mosaic virus (CaMV) 35S promoter was transiently expressed in living onion epidermal cells. As shown in Figure 3, confocal microscopic examination revealed that cells that transiently expressing unconjugated GFP (control) exhibited a diffuse distribution of green fluorescence throughout the cell (Figure 3A-B). By contrast, the signal generated by cells that transiently expressing Hv-SGT1::GFP was confined to the cytoplasm and nucleus (DAPI stained) of onion epidermal cells (Figure 3C-D), suggesting that *Hv-SGT1* is restricted to the cytoplasmic and nuclear compartments. Similar differences in the subcellular locations of the two constructs were evident following transient expression of GFP and the Hv-SGT1::GFP fusion in leaf epidermal cells of *H* *. villosa* (Figure 3E and 3F).

**Figure 4:**
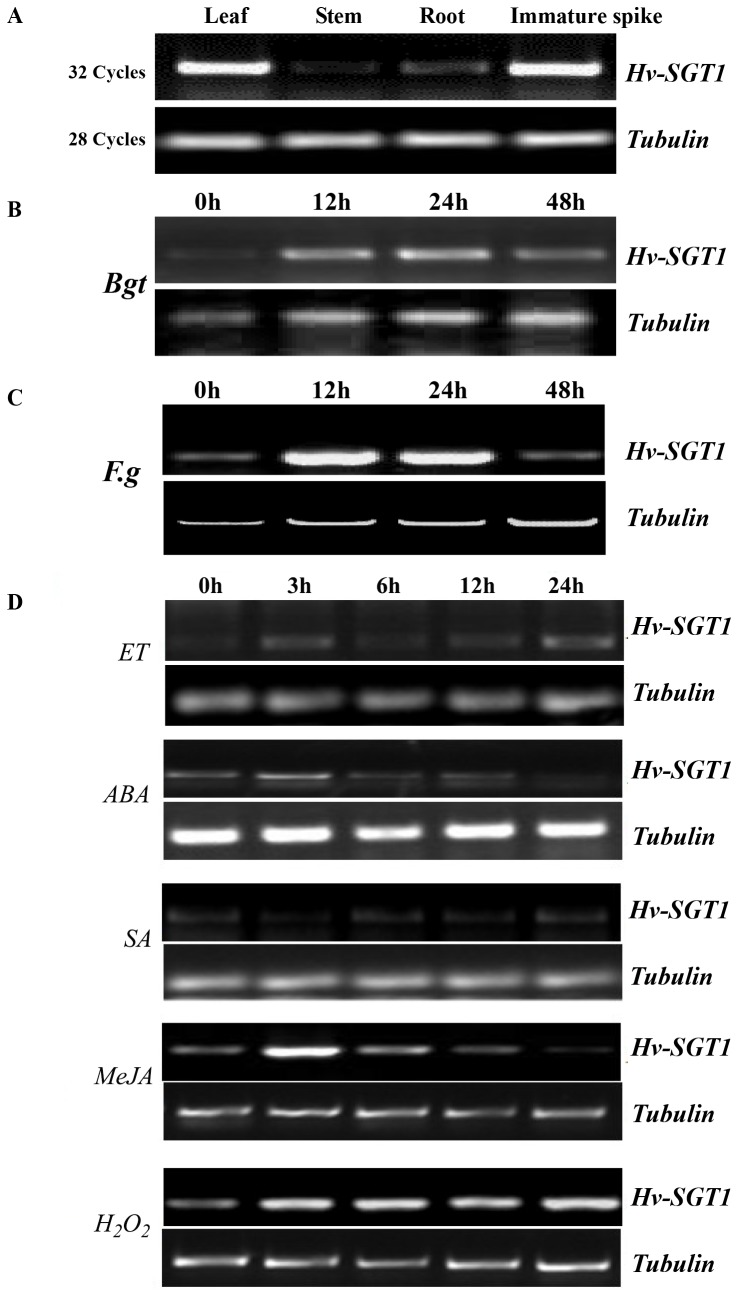
Responses of *SGT1* to treatments of biotic and abiotic stresses, as well as phytohormone applications. Expression patterns of *SGT1* (A) in different organs of *H* *. villosa* , (B) in *H* *. villosa* leaves after *Bgt* inoculation, (C) in *H* *. villosa* immature spikes after *Fg* inoculation, and (D) in *H* *. villosa* leaves fter treatments with ET, ABA, SA, MeJA, and H_2_O_2_.

Expression profiling of *Hv-SGT1* using different tissues of *H* *. villosa* showed that the highest expression was observed in leaves and immature spikes, with lower levels of expression observed in stems and roots (Figure 4A). Expression of the *Hv-SGT1* gene was rapidly induced during infection of the leaves of seedlings of *Bgt*-resistant *H* *. villosa* , with levels of transcripts peaking 12 h after infection (Figure 4B). Similar inducible expression patterns were also observed in the immature spike of *H* *. villosa* after infection with *Fg* (Figure 4C). Treatment of the leaves of *H* *. villosa* seedlings with different phytohormones revealed that *Hv-SGT*1 transcripts were strongly induced by H_2_O_2_ and methyl jasmonate (MeJA), slightly induced by ethepon (ET) and abscisic acid (ABA), but not by salicylic acid (SA) (Figure 4D). The inducible expression of *Hv-SGT1* following pathogen infection and treatment with phytohormones suggested its involvement in disease resistance reactions and defense pathways.

### Knockdown of *Hv-SGT1* by barley stripe mosaic virus–induced gene silencing in *H. villosa* enhances susceptibility of the host to *Bgt*

Barley stripe mosaic virus induced gene silencing (BSMV-VIGS) has been used to characterize gene function in wheat and *H* *. villosa* [24]. To confirm the role of *Hv-SGT1* in the defense response of *H* *. villosa* to *Bgt* infection, we performed VIGS of *Hv-SGT1* in *H* *. villosa* .

**Figure 5:**
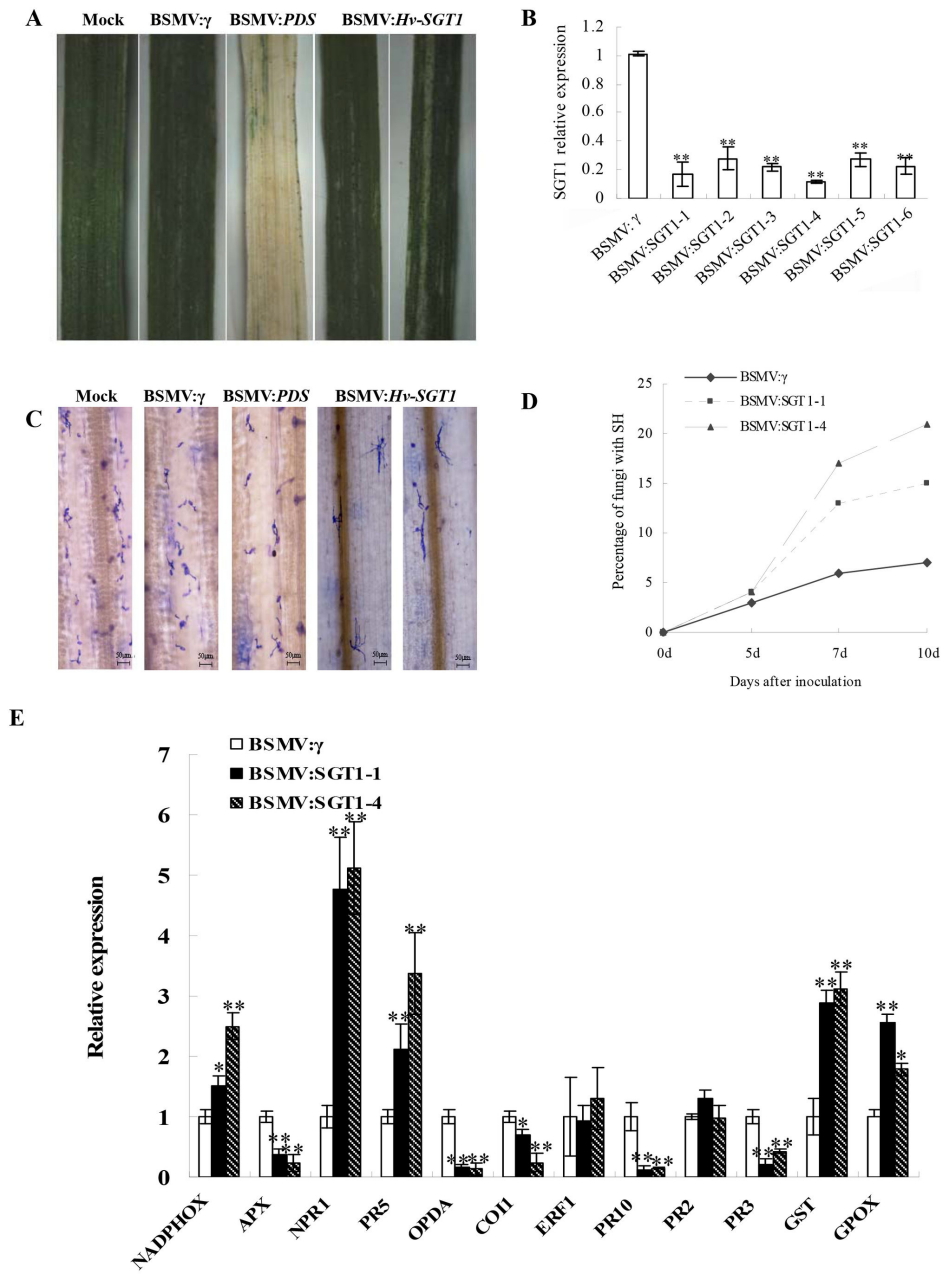
Functional analysis of the *Hv-SGT1* gene by BSMV-induced gene silencing. (A) Following infection of *H* *. villosa* with the virus, chlorotic mosaic symptoms were observed on the 4^th^ leaves 10 days after inoculation (dpi) with BSMV:γ or BSMV:*Hv-SGT1*. Photobleaching was observed on leaves infected with BSMV:*PDS* 15 dpi, whereas no detectable phenotype was observed in the mock-treated plants inoculated with 1×GKP buffer. Representative photographs were taken 15 days after virus inoculation. (B) The *Hv-SGT1* gene was efficiently silenced in the BSMV: Hv-SGT1 inoculated leaves compared to the control inoculated with BSMV:γ, as indicated using qRT-PCR analysis. (C) More infected *Bgt* could developed into secondary hyphae in the BSMV: Hv-SGT1 inoculated leaves compared to the BSMV:γ infected leaves 10 days after inoculation with *Bgt*. (D) The rate of formation of secondary hyphae (SH) was higher in the *Hv-SGT1* silenced leaves inoculated with BSMV:Hv-SGT1 compared with that in BSMV:γ-infected leaves at 5, 7, and 10 days after inoculation with *Bgt*. (E) Levels of *NADPHOX*, *APX*, *NPR1*, *PR5*, *OPDA*, *COI1*, *ERF1*, *PR10, PR2, PR3, GST*and *GPOX*in *Hv-SGT1* silenced leaves. * p < 0.05, ** p < 0.01 compared to the BSMV:γ-infected leaves.

First, checking the efficiency of the VIGS system revealed that mild chlorotic mosaic symptoms were observed on the fourth leaves inoculated with BSMV at 10 days after inoculation. Photobleaching was observed on leaves infected with BSMV:*PDS* at 15 days, whereas no obvious changes were observed for mock leaves inoculated with 1×GKP buffer (Figure 5A). The level of expression of *Hv-SGT1* was checked by qRT-PCR and found to decrease dramatically in *H* *. villosa* plants inoculated with BSMV:*Hv-SGT1* compared with the *Bgt* inoculation plants preciously infected with BSMV: γ (Figure 5B).

The VIGS data indicated that 10 days after inoculation with *Bgt*, only the primary germ tubes (pp) and appressorium penetration peg (app) could be detected for most geminated spores (Figure 5C), and fewer than 5% of all spores could produce secondary hyphae (SH) in *H* *. villosa* plants that either received mock inoculation or inoculation with BSMV:γ. However, for those BSMV:*Hv-SGT1* treated leaves, 15–20% of the spores could produce SH and even conidial chains (Figure 5D). The evidence that the resistance of *H* *. villosa* to *Bgt* was compromised when *Hv-SGT1* was silenced underscores the notion that *Hv-SGT1* is essential for the resistance of *H* *. villosa* to powdery mildew.

Given that *Hv-SGT1* expression was phytohormone-dependent and related to disease resistance, we next examined the expression of a set of genes associated with the H_2_O_2_, SA, and JA pathways in the *Hv-SGT1*-silenced plants. Whereas levels of the transcript that encoded NADPH oxidase (NADPHOX, a H_2_O_2_-producing gene product) were elevated in the *Hv-SGT1*-silenced plants, levels of the transcript that encoded APX (a H_2_O_2_-scavenging gene product) were lower in *Hv-SGT1*-silenced plants than in control plants, while the transcript levels of both GST and GPOX (a ROS-scavenging gene product) were higher, revealing close link between SGT1 and the activity of antioxidative protection system. Transcript levels of *NPR1* and *PR5*, which mediate the SA-related defense pathway [25], were significantly higher in *Hv-SGT1*-silenced plants than those in the control. However, the association of the transcription level of *PR2* (marker gene of the SA pathway) with *SGT1* was not observed. The transcript levels of *PR3* (marker gene of the ET pathway) were significantly lower in *Hv-SGT1*-silenced plants than that in the control. Investigation of transcripts rrelated to the JA-mediated defense signaling (*OPDA*, *COI1*, *ERF1*, and *PR10*) [26,27], revealed a substantial reduction in levels of *OPDA*, *COI1*, and *PR10* transcripts in *Hv-SGT1*-silenced plants, but no change in expression of *ERF1* (Figure 5E). These results implied that *Hv-SGT* was intimately related to phytohormone-dependent defense pathways.

### Over-expression of *Hv-SGT1* enhances the resistance of common wheat to the biotrophic pathogen *Bgt*

**Figure 6:**
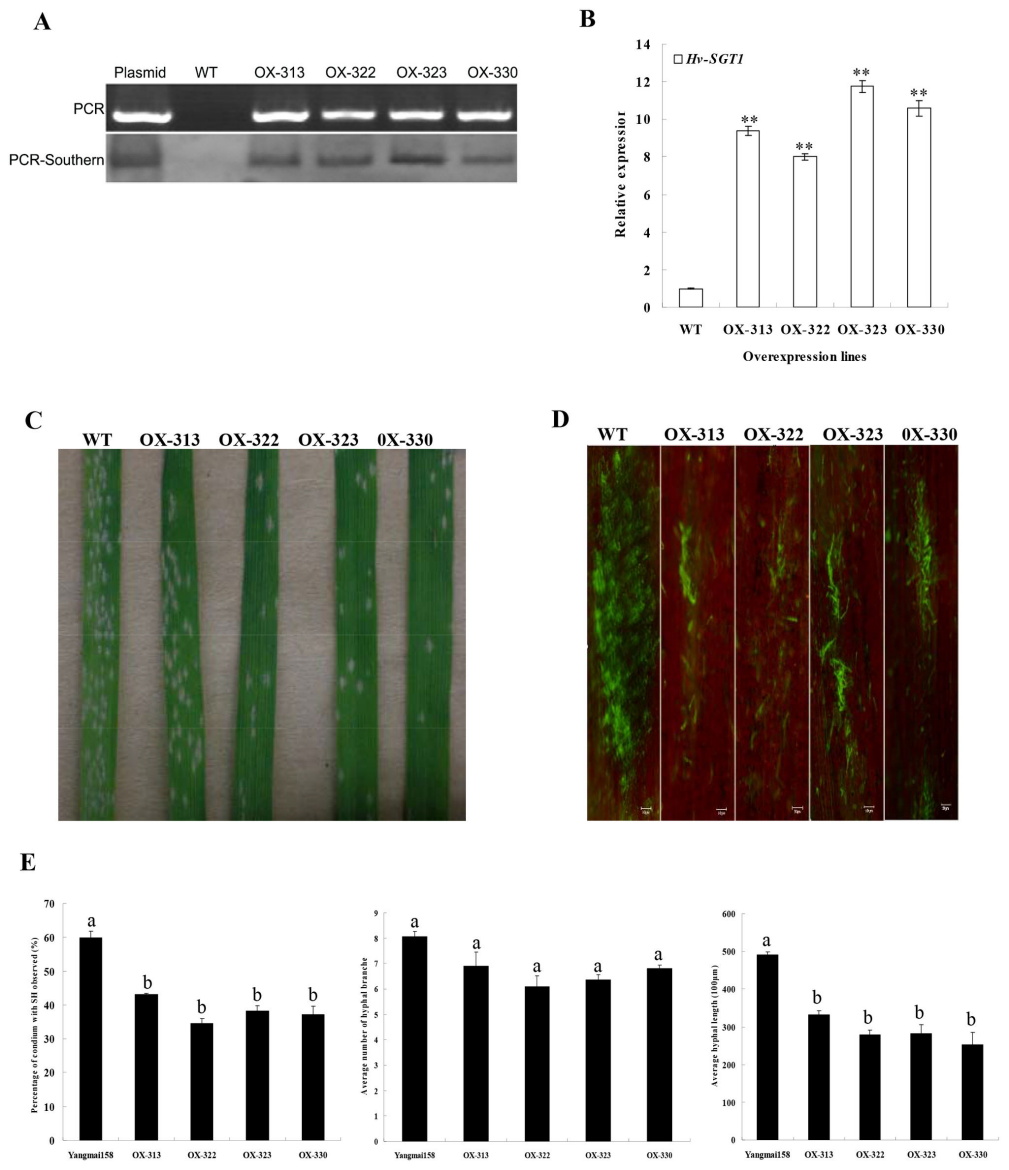
Characterization of the transgenic wheat of *Hv-SGT1*. (A) PCR and PCR-Southern blot of four transgenic lines that over-expressing *Hv-SGT1* (OX), and the non-transformed control Yangmai 158. The plasmid *pBI220.6-Hv-SGT1* and Yangmai 158 were used as the positive and negative controls, respectively. (B) qRT-PCR of the expression of *Hv-SGT1* in the four transgenic lines and Yangmai 158. ** p < 0.01 compared with the control. (C) Reduced disease symptoms in transgenic plants. Seedling resistance of transgenic line or wild-type plants was assessed following *in vitro* infection with the native pathogen population (Sumai 3). (D) Microscopic observation of *Bgt* hyphae spreading after DioC6 staining of the transgenic plants and Yangmai 158. (E) Quantitative comparisons of the percentage of infection sites with secondary hyphae (SH), the average number of hyphal branches and average hyphal length emerging on the leaves of infection sites. Means (± SE) were calculated using the measurements from five seedlings, and at least 30 infection sites for each seedling. Significance was determined according to paired sample *t*-test method (b indicates *P* < 0.05).

Although it has been well documented that SGT1 regulated defense responses triggered by various pathogens, less research had investigated its effectiveness to both biothophic and hemi-biothophic pathogens. Transgenic Yangmai 158 plants that over-express *Hv-SGT1* under the regulation of the CAMV35S promoter were obtained by using the particle bombardment-mediated transformation approach. Overall, 52 independent T_0_-generation transgenic plants were identified after analysis of 432 regenerants using PCR amplification. Four of these lines (OX-313, OX-322, OX-323, OX-330) were characterized by further Southern blot of the PCR products (Figure 6A). qRT-PCR of the identified four lines indicated that expression of *Hv-SGT1* in the transgenic plants was significantly increased up to 7.5-fold compared with that in the non-transformed Yangmai 158 (Figure 6B). The progenitors derived from these four positive transgenic plants were analyzed to assess their resistances to powdery mildew.

Compared with the wild type (WT) control Yangmai 158, transgenic T_2_ plants that over-express *Hv-SGT1* (a total of 151 individuals were derived from the four T_0_ plants mentioned above) showed enhanced resistance both in the seedling and adult stages (Table 1, Figure 6C), and the development of *Bgt* was observed under the microscope. The area of *Bgt* spreading at the infection sites was much smaller in the transgenic plants than that in Yangmai 158 (Figure 6D). It was proposed that the increased resistance of the transgenic plants was related to the effective suppression of the hyphal development after successful penetration. The percentage of infection sites with secondary hyphae (SH), the average number of hyphal branches and average hyphal length at the infection site were investigated by histological observations at 48h after *Bgt* inoculation. A significantly (*P* < 0.05) lower percentage of infection sites producing SH (37.1%-43.1%) and shorter hyphal length (25,700-34,600 µm) at infection sites were observed in transgenic plants compared with corresponding values of 59.9% and 48,900 µm in Yangmai158. However, there was no significant difference in number of hyphal branches between control and transgenic plants (Figure 6E).

**Table 1:** *Bgt* responses of *Hv-SGT1* transgenic plants and Yangmai158.

Lines	No. of T_2_ positive plants	Disease Index of T_2_ generation		Significance of ANOVA* (p<0.05)	Response ^a^
		Seedling	Adult		
OX-313	36	52.47+18.52	47.84+11.52	B	MR
OX-322	38	61.70+24.33	54.09+16.78	B	MS
OX-323	47	54.61+21.71	47.52+14.60	B	MR
OX-330	30	62.22+22.90	52.22+14.92	B	MS
Yangmai 158	50	91.33+7.54	92.22+6.44	A	HS

^a^ Means different responses to *Bgt*. HS: highly sensitive; MS: moderately sensitive; MR: moderately resistant. ^*^ Means with the same letter are not significantly different.

**Figure 7:**
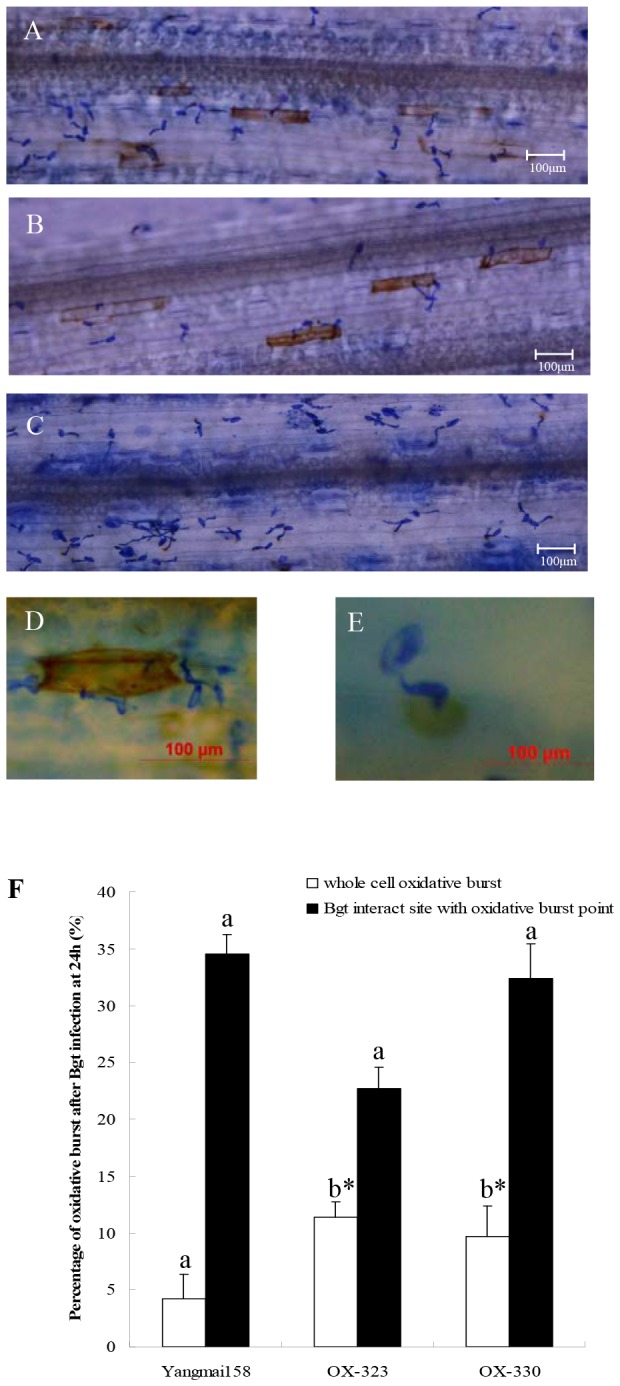
Hydrogen peroxide accumulation in leaves of *Hv-SGT1* over-expressing plants and the WT Yangmai 158. Hydrogen peroxide accumulated in Yangmai 158 (A), and *Hv-SGT1* over-expressing lines OX-323 (B) and OX-330 (C). (D) Whole-cell ROI accumulation. (E) Oxidative burst at the *Bgt* interaction site. (F) Comparison of the percentage of cells with H_2_O_2_ accumulation throughout the entire cell or only around the infection sites in wild-type Yangmai 158 and the transgenic plants (* means p < 0.05).

Histological examination revealed that lines that over-expressed *Hv-SGT1* showed a higher frequency of *Bgt*-induced whole-cell oxidative burst. Staining with 3, 3′-diaminobenzidine (DAB), which allows detection of H_2_O_2_, did not uncover genotype-specific differences at an early stage of the interaction (12 h post inoculation [hpi], data not shown). At 24 hpi, the fungus had penetrated epidermal host cells in up to 60% of interaction sites in wild-type (WT) plants as well as in the transgenic lines OX-323 and OX-330. Detection of DAB polymerization showed a similar whole-cell accumulation ratio of H_2_O_2_ in both OX-323 and OX-330 (Figure 7A and 7B). In contrast, only a few DAB-stained cells (Figure 7D) was detected in WT Yangmai 158, although appressorial germ tube (AGT) penetration sites and primary germ tube (PGT) penetration sites with oxidative burst points were observed (Figure 7C). More ROIs accumulated in most pathogen-inoculated transgenic leaves (11.4% in OX-323, 9.7% in OX-330) than in the inoculated Yangmai 158 leaves (4.2%), although there was no significant difference in the frequencies with which oxidative burst points formed at *Bgt* interaction sites (Figure 7E) in leaves from WT, OX-323, or OX-330 plants (Figure 7F). These revealed that the pathways responsible for the oxidative burst were activated for the host defense response though regulation of *Hv-SGT1*.

**Figure 8:**
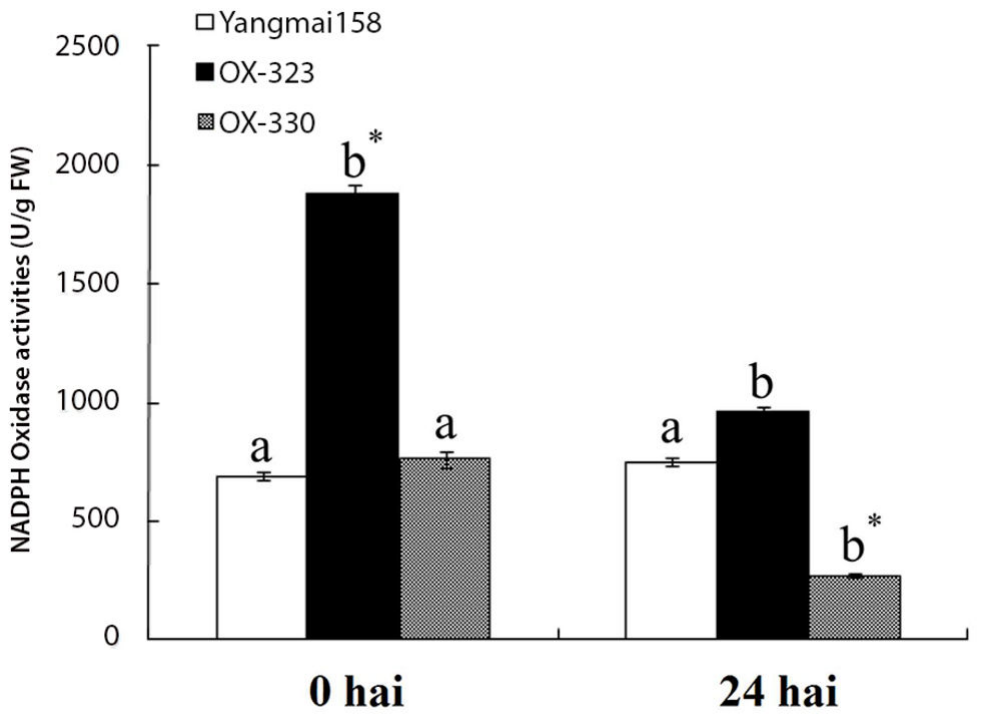
NADPH-_OX_ activities in transgenic and WT plants. Each point represents the mean of three replicates. Bars indicate ±SE, Mean values followed by different letters are significantly different from each other (b* indicates P < 0.01 and b indicates P < 0.05).

Plant NADPH oxidasetive (NADPH-_OX_) enzyme was called Rboh (respiratory burst oxidase homologue), rapidly increases or decreases reactive oxygen species (ROS) through self-activation or inactivation during plant responses to biotic stresses. Two *Hv-SGT1* transgenic lines OX-323, OX-330, and the Yangmai158 (WT) control, were analyzed for NADPH-_OX_ activities using fully expanded leaves. NADPH-_OX_ activities in the two transgenic lines were enhanced by 173.9% and 10.2% before *Bgt* inoculation (0 hai), but were decreased at 24 hai, respectively, in contrast to WT plants (Figure 8). These results suggested that varied NADPH-_OX_ activities in the transgenic lines were likely associated with expression of *Hv-SGT1*.

### Over-expression of *Hv-SGT1* contributes to the resistance of common wheat to the hemi-biotrophic pathogen *Fg*

**Figure 9:**
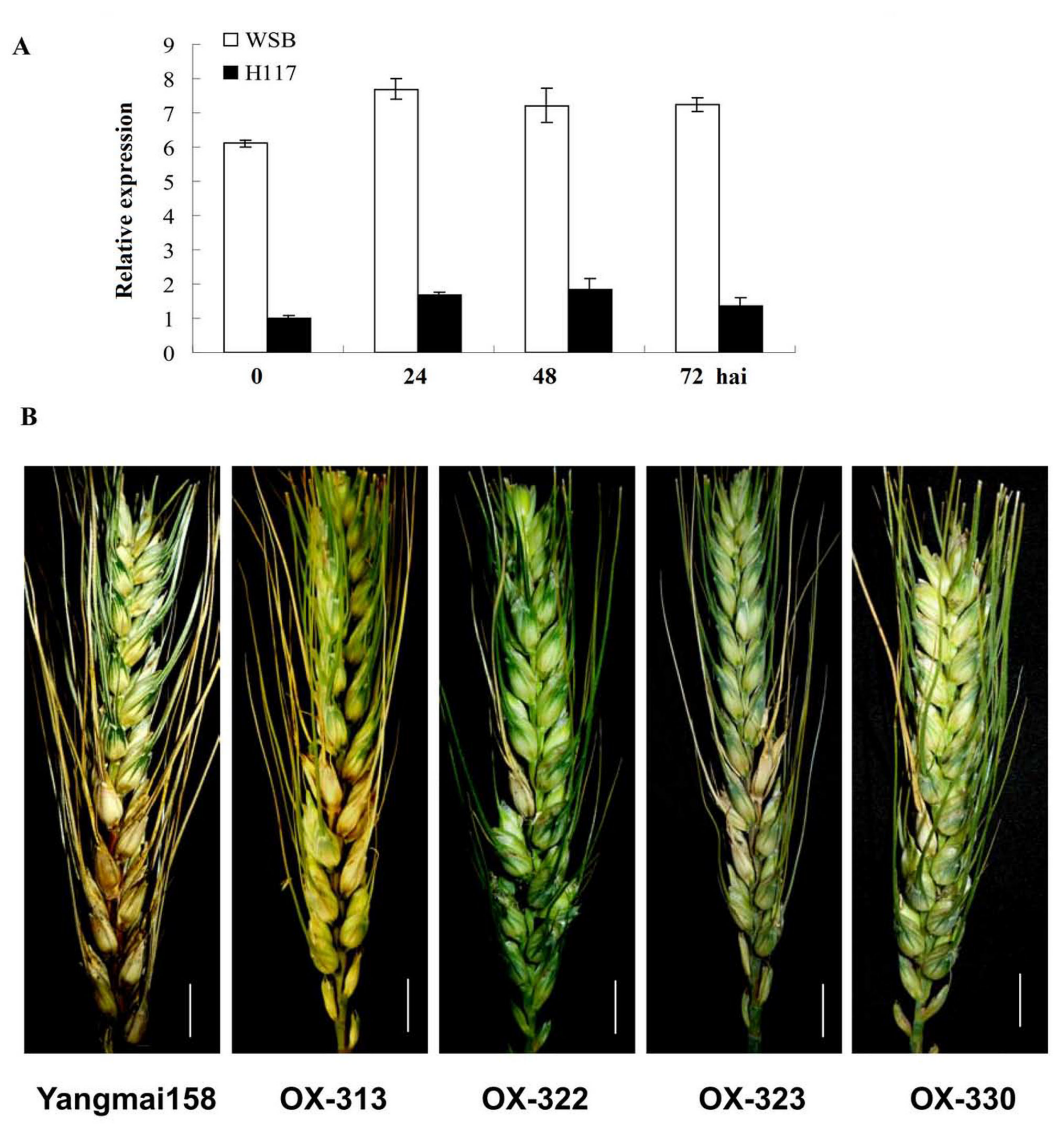
Relative expression levels of *Ta-SGT1* in spikes of scab-resistant wheat variety Wangshuibai (WSB) and its susceptible mutant NAUH117 at different times after inoculation with *Fg*. (A) *Ta-SGT1* was constitutively expressed in both WSB and NAUH117, and no obvious change was detected after inoculation with *Fg*. Nonetheless, an approximately 6-fold lower expression level of *Ta-SGT1* transcript was detected in the susceptible mutant NAUH117 than that in resistant WSB. (B) The FHB symptom of spikes of Yangmai158 and transgenic plants at 21 days after *Fusarium* inoculation, scale bar represents 1cm.

Unlike the inducible expression pattern of *Hv-SGT1* in spikes of *H* *. villosa* after inoculation with *Fg*, *Ta-SGT1* was constitutively expressed in both the FHB-resistant Wangshuibai (WSB) cultivar and its susceptible mutant NAUH117 (Figure 9A), and no obvious change was detected after inoculation with *Fg*. Nonetheless, an approximately 6-fold lower expression level of *Ta-SGT1* transcript was detected in the susceptible mutant NAUH117 than that in resistant WSB. To determine whether higher expression levels of *SGT1* was required for the resistance to *Fg*, we further investigated the over-expression plants transformed to express *Hv-SGT1* under the regulation of the CAMV35S promoter for resistance to infection by the hemi-biotrophic *Fg.*

To evaluate resistance to FHB, transgenic T_1_ (164 individuals derived from the four positive T_0_ plants during 2010–2011) and T_2_ (151 individuals derived from the four positive T_0_ plants during 2011–2012) plants were grown under both greenhouse and field conditions, respectively. For both T_1_ and T_2_ plants, their average percentages of diseased spikelets were significant reduced compared with those of Yangmai 158 (Figure 9B, Table 2). This indicated that over-expression of *Hv-SGT1* also contributed to the enhanced resistance of the transgenic plants to wheat FHB.

**Table 2:** Average rate of scab diseased spikes of the T_1_ and T_2_ transgenic plants.

Lines	No. of positive plants	Average rate of scab diseased spikes		Significance of ANOVA* (p<0.01)
		T_1_ generation	T_2_ generation	
OX-313	20	6.7±2.6	6.0±1.6	B
OX-322	20	4.5±2.4	5.8±0.1	B
OX-323	20	5.8±2.3	5.9±1.3	B
OX-330	20	7.4±1.1	6.3±1.2	B
Yangmai 158 ^a^	5	8.4±2.7	15.8±5.1	A
Sumai 3 ^b^	5	2.5±0.1	4.5±0.5	B
Mianyang 85-45 ^c^	5	20.6±5.5	24.0±3.2	A

^a^ Yangmai 158 is the parental control; ^b^ Sumai 3 and ^c^ Mianyang 85-45 are the disease resistant and susceptible controls, respectively. ^*^ Means with the same letter are not significantly different.

### *Hv-SGT1* over-expression regulates the expression of genes related to both the H_2_O_2_ and JA pathways

Given our demonstrations that both H_2_O_2_ and JA could induce the expression of *Hv-SGT1*, and that over-expression of *Hv-SGT1* resulted in H_2_O_2_ accumulation in transgenic plants after inoculation with *Bgt*, we deduced that and H_2_O_2_ and JA might be linked to the expression of *Hv-SGT1*. The SA pathway is also closely related to plant disease resistance, and has been associated with HR induced by H_2_O_2_. We therefore analyzed the expression patterns of a subset of genes related to producing or scavenging H_2_O_2_, as well as genes related to SA- and JA-mediated signaling.

**Figure 10:**
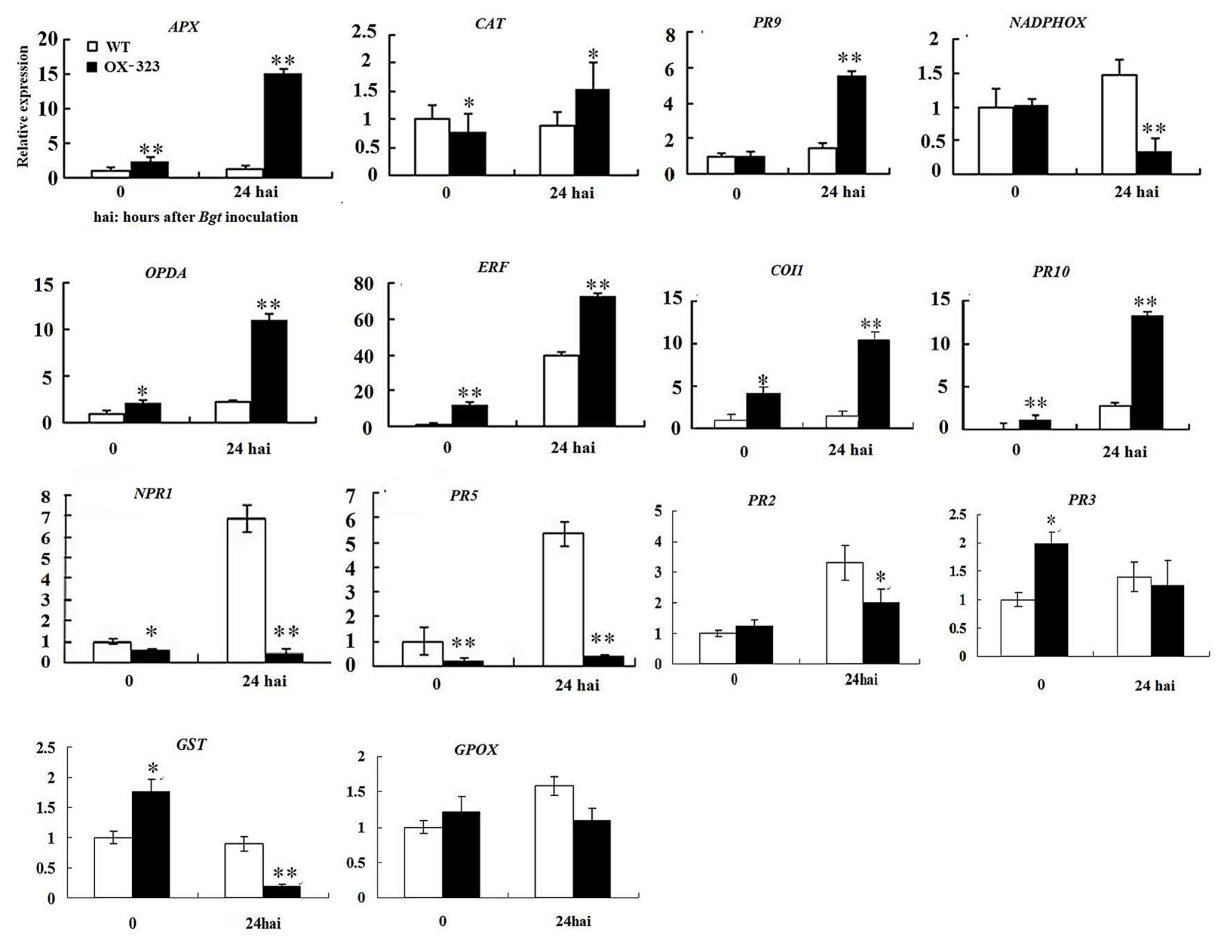
Real-time qPCR analysis of the expression patterns of ten genes related to pathogenesis pathway in the wild-type Yangmai 158 and *Hv-SGT1-*over-expressing transgenic plants (OX-323) before inoculation of *Bgt* and 24 h post inoculation with *Bgt*. * p < 0.05, ** p < 0.01 compared with the wild type. Over-expression *Hv-SGT1* regulates the expression of genes related to the H_2_O_2_, SA and JA pathways.

The H_2_O_2_-producing gene *PR9* was significantly up-regulated in the transgenic plants after *Bgt* inoculation. Interestingly, another gene responsible for H_2_O_2_ production, *NADPHOX*, was down-regulated following inoculation with *Bgt*. The H_2_O_2_-scavenging gene, *APX*, was significantly up-regulated in the transgenic plants after inoculation with *Bgt*, and the *CAT* gene, which has a similar function, was only slightly up-regulated. Unlike enzymatic antioxidative system genes, *GST* (marker gene of non-enzymatic antioxidative system) was significantly down-regulated in the transgenic plants after inoculation with *Bgt*, but the *GPOX* gene, which has a similar function, was not regulated by *SGT1* (Figure 10). After *Bgt* inoculation, the expression levels of genes related to the JA-signaling pathway, including those involved in JA synthesis (*OPDA*), JA signal transduction (*COI1* and *ERF*), and response to the JA signal (*PR-10*) were substantially higher (Figure 10) in OX-323 transgenic plants than in WT plats after inoculation with *Bgt*. However, expression of marker genes of the SA pathway, such as *NPR1*, *PR5* and*PR2*, was higher in the transgenic plants than in the WT plants, but down-regulated after inoculation with *Bgt* (Figure 10). Meanwhile, we found expression of *PR3* (marker gene of ET pathway) was hardly changed in the transgenic plants both before and after inoculation with *Bgt*. We presumed that the over-expression of *Hv-SGT1* might regulate the balance between the production and scavenging of H_2_O_2_, stimulate JA signaling, and suppress the SA defense pathway, but was not directly involved in the ET defense pathway.

## Discussion

This study characterized the induction of the *Hv-SGT1* gene by both biotrophic and hemi-biotrophic pathogens. Homology and cluster analyses indicated that *Hv-SGT1* belongs to the SGT protein family found in eukaryotes, and that the homologies of SGTs from various organisms are relatively high. The main similarities between SGT proteins lie in the conserved TPR domain and SGS motifs, although the considerable diversity in the CS region, which was required for its binding to HSP90 [28], suggested that the functions of *Hv-SGT1* may differ from other SGTs. At the subcellular level, *Arabidopsis* *SGT1b* fused to Cerulean localized to the cytosol but could be seen in nuclei of 25% of 55 transformed cells examined. This suggested the movement of SGT1b between the cytosol and nucleus [29]. Moreover, a fusion of rice OsSGT1 to GFP localized to both the cytoplasm and nuclei of onion epidermal cells [13], which was consistent with our localization of Hv-SGT1-GFP fusion protein. This suggested that the rice and Arabidopsis SGT1 proteins might have similar functions associated with plant disease resistance.

Microarray analysis identified *Hv-SGT1* as a candidate gene that regulates broad-spectrum resistance to *Bgt* in *H* *. villosa* . However, it is challenging to generate α Bgt susceptible mutant or *SGT1* knockout mutant of *H* *. villosa* , and although immature embryo culture and regeneration system for transformation were established for wheat, these approaches were not yet available for *H* *. villosa* . Therefore, the goal of this research was to demonstrate stable constitutive expression of *Hv-SGT1* in Yangmai 158, a wheat variety with moderate susceptibility to both powdery mildew and scab.

As an ubiquitylation pathway component, *SGT1* plays important roles in the plant defense. Unver et al. [30] found that mRNA levels of *SGT1* was up-regulated following *NbUbc2* silencing. SGT1 positively regulated the disease resistance via interaction with the UBC2 or ubiquitylation in *Nicotiana* *benthamiana* . Similar results were also obtained in *TaUbc2*-silenced wheat leaves [31]. Our results were consistent with the above results, showing that *HvSGT1* was also a positive regulator in the plant hypersensitive response, although we haven’t found its interaction substrate or UBC in this paper.

The interplay among complex signaling networks, including various pathways regulated by phytohormones such as salicylic acid (SA), jasmonic acid (JA), ethylene and ABA, dramatically influences plants’ disease resistances. By precisely regulation of the accumulation and perception of phytohormones, plants counteract disease stresses. In recent years, the biosynthesis, signal transduction, and physiological functions of phytohormones in various plant species have been better understood. The roles of SGT1 in plant disease resistance, especially its link with phytohormone pathways have been intensively studied. Meldau et al. [15] found that SGT1 was required for herbivory-induced SA and JA homeostasis and normal MeJA-induced transcriptional responses, and was important for the biosynthesis of secondary metabolites defense responses against phytophagous insects in *N* *. attenuate* . Our results further confirmed the involvement of SGT1 in different phytohormone signaling (H_2_O_2_, JA and SA) both in *H* *. villosa* and wheat. Further characterization of the involvement of SGT1 in defense responses mediated by the phytohormone pathways will help us to unravel the roles of *Hv-SGT1* in powdery mildew resistance of wheat.

Several previous studies have shown that *SGT1* is required for cell death during incompatible interactions associated with HR [3,8,32,33]. The accumulation of H_2_O_2_ accumulation is believed to be involved in HR during plant-pathogen interactions. A previous study demonstrated that a serine/threonine kinase gene *Stpk-V*, which is a key member of the broad-spectrum resistance locus, can regulate H_2_O_2_ accumulation in the wheat-*Bgt* interaction. In this study, we demonstrated differential expression of both H_2_O_2_-producing and H_2_O_2_-scavenging genes in the *SGT1* transgenic wheat and the parental line from which these were derived. We observed a far more obvious accumulation of H_2_O_2_ after inoculation of *Bgt* in the transgenic plants compared with the WT line from which they were derived. This indicated that the increased H_2_O_2_ level was correlated with the over-expression of *SGT1*. The accumulation of H_2_O_2_ and the subsequent cell death usually resulted in the disease resistance caused by the biothophic pathogens [34]. Over-expression of *NbSGT1* accelerates cell death associated with resistance to a non-adapted pathogen [8], although over-expression of *SGT1* in rice did not enhance disease-associated cell death [13]. It was proposed that different *SGT1*-pathogen interactions and the complexity of the cell death pathways determine whether cell death can be induced by the over-expression of *SGT1*. But expression levels of *NPR1* and *PR5*, which could be accounted for the component of SA pathway activity, were decreased after *Bgt* inoculation when the cellular H_2_O_2_ content was increased. We supposed that *SGT1* positively regulated the H_2_O_2_ pathway, meanwhile negatively regulated the SA pathway. Moreover, H_2_O_2_ might not directly transfer the SA signal to the downstream response genes in the *SGT1* transgenic plants.

In *Arabidopsis* , the CWP (cell wall protein fraction)-induced defense system appeared to be regulated by JA-mediated and *SGT1*-dependent signaling pathways [35]. What role does the JA pathway play in *Hv-SGT1-*mediated disease resistance in wheat? The enzyme 12-oxophytodienoate reductase (OPDA) plays a key role in the biosynthesis of JA [36]. *Arabidopsis* *SGT1b* is also required for the activities of the SCF^COI1^ -mediated JA response [37,38]. The COI1 protein contains an F-box motif and associates physically with AtCUL1, AtRbx1, and the Skp1-like proteins to assemble SCF^COI1^ ubiquitin-ligase complexes for ubiquitination and subsequent degradation by the 26S proteasome [39,40]. The JA-responsive gene that encodes ethylene response factor (ERF) plays a crucial role in the cross-talk between JA and ET signal transduction [26,41]. The *PR10* gene have been implicated in the signaling pathway that responds to JA, and is critical to effective functioning of the JA defense pathway [42]. In our research, expression of genes involved in JA signaling and response pathways was enhanced in *Hv-SGT1* transgenic plants and in *Hv-SGT1*-silenced plants. Expression of *NPR1*, *PR2* and *PR5*, which are markers of SA pathway activity, was either reduced in *Hv-SGT1*-overexpressing plants after *Bgt* inoculation or enhanced in *Hv-SGT1*-silenced plants. Differently, expression level of *PR3* was unobviously changed in the transgenic plants both before and after inoculation with *Bgt*. This suggests that the transactivation of *PR* genes in SA pathway was independent of *SGT1*, and *SGT1* was not directly involved in the ET defense pathway to regulate the *PR3* expression. We propose that *Hv-SGT1* activates resistance mechanisms through JA-dependent defense pathways, but suppresses the activities of SA-dependent defense pathways, and does not affect the ET-dependent defense pathways.

EI Oirdi & Bouarab [19] demonstrated that silencing *SGT1* in *N* *. benthamiana* compromises the hypersensitive response induced by *Botrytis* *cinera* . They hypothesized that *B* *. cinera* promotes *NbSGT1* expression to exploit the antagonistic effects between SA and JA. Higher basal concentrations of SA were also found in *NaCOI1*-silenced *N* *. attenuata* , suggesting that impaired JA signaling increases SA concentrations [43]. It is not enough to detect the transcription level of pathway-related genes only. Whether SGT1 also mediates the homeostasis of pathogen-elicited SA and JA levels by measurement changes of the SA and JA in both *SGT1*-silenced and *SGT1*-overexpressing plants, deserves further study.

There is compelling evidence that *SGT1* contributes to the resistance to powdery mildew mediated by race-specific *R* genes in barley [18]. In the present study, both the VIGS and gene transformation experiments proved that *SGT1* was tightly correlated with the powdery mildew resistance in *H* *. villosa* , which showed substantial broad-spectrum resistance to powdery mildew. The increased cell death due to the over-expression of the *SGT1* and the activated JA pathway might both contribute to the improved resistance. Our results indicated that *SGT1* was also related to the observed broad-spectrum resistance, but the role of *SGT1* in broad-spectrum disease resistance was still poorly understood. Further analysis of the function of *SGT1* will enhance our understanding of the roles of *SGT1* in the interaction of *H* *. villosa* with *Bgt*.

There are differences in the resistance mechanisms and patterns of fungal development seen in biothophic and necrotrophic pathogens, and *Fg* is a hemi-biotrophic pathogen with both biotrophic and necrotrophic features. The earliest stages of *Fg* infection are characterized by biothophic features, although the pathogen proceeds to the necrotrophic phase after successful infection. The SA pathway is critical for scab resistance at the early stage of infection, when *Fg* attacks the host as a biothroph, although the JA pathway plays a predominant role at later stage when the pathogen develops necrotrophic tendencies [44]. The SA pathway is usually correlated with HR and cell death, which effectively restricts infection by the biothophic pathogen. A study conducted using tobacco indicated that *SGT1* is responsible to cell death, and SGT1 was a positive regulator of the JA pathway [15]. In this study, the over-expression of *SGT1* in wheat variety Yangmai 158 can increase its resistance to *Fg*, which may be the result of the increased cell death caused by over-expression of *SGT1* at the site of primary infection. The activated JA pathway in the transgenic wheat may contribute to the resistance to the *Fg* during a later stage of infection.

Previous studies have showed that SGT1 plays an important role in the resistance to necrotrophic pathogens. For example, SGT1 of *Nicotiana* *benthamiana* contributed symptom development and disease susceptibility after infection by the necrotrophic fungus *Botrytis cinerea* [19]. However, our research has implied an important role for SGT1 in this hemi-biotrophic pathogen. Although our preliminary results showed that the transgenic plants showed increased resistance to the *Fg*, further experiments (including gene silencing) should be performed using a resistant variety, such as Sumai 3 or Wangshuibai, to clarify the involvement of *SGT1* in resistance to *Fg*.

Some studies have demonstrated an essential role for *SGT1* in basal resistance to phytopathogens. For example, over-expression of *OsSGT1* from rice significantly increases basal resistance to the virulent bacterial blight pathogen *Xanthomonas* *oryzae* pv. *oryzae* (Xoo) strain *PXO99* and to several blast fungal *M. grisea* races [13]. Many studies also implied a role for SGT1 in *R*-gene-mediated resistance. Although SGT1 is associated with resistance to both *Bgt* and *Fg* in wheat, it remains unknown whether it functions in the PAMP-triggered basal resistance stage or in the effector-triggered resistance stage.

Owing to the different (sometimes even contradictory) mechanisms of resistance to biotrophic and necrotrophic pathogens, the co-regulation of the resistance to both classes of pathogens seems to be complicated. The co-regulation of resistances to the bithophic *Bgt* and the hemi-biotrophic *Fg* demonstrated in the present study indicates the feasibility of improving the resistance to different types of pathogens if the universal regulatory factors are explored and used properly. Our results provide further evidence for the universal role of the *SGT1* gene in various plant defense responses. Over-expression of *Hv-SGT1* conferred significantly increased resistance to wheat powdery mildew and scab, providing an effective strategy for developing wheat germplasm with improved resistance to the pathogens responsible for these diseases.

## Materials and Methods

### Plant materials

*Haynaldia* *villosa* (2n=14, VV), *T* *. durum* - *H* *. villosa* amphiploid (AABBVV), DA1V-DA7V (*T. aestivum*- *H* *. villosa* addition line, each contains one pair of chromosomes of *H* *. villosa* from 1V to 7V in the common wheat background), powdery mildew susceptible wheat varieties Chinese spring and Yangmai 158, powdery mildew resistant wheat variety *T. aestivum–H. villosa* T6VS/6AL translocation line 92R137, FHB resistant wheat variety Wangshuibai to and its susceptible mutant NAUH117 were all developed or preserved by Cytogenetic Institute, Nanjing Agricultural University (CINAU).

### Fungal and chemical treatments

Mixed races of *Bgt* were maintained on susceptible variety Sumai 3 seedlings in a spore-proof greenhouse under 14 h light/10 h dark (24/18°C, 70% humidity) regime. Wheat seedlings at the two-leaf stage were inoculated with *Bgt* or treated with exogenous hormone or signal molecules, including 5 mM salicylic acid (SA), 100 μΜ methyl jasmonate (MeJA), 200 μΜ ethepon (ET), 5 μΜ abscisic acid (ABA), and 7 mM hydrogen peroxide (H_2_O_2_). All chemicals were administered as a 0.05% Tween-20 solution, with 0.05% Tween 20 used as a mock treatment, and all of the treatments followed Cao et al. [22]. Leaves were harvested at different time points after treatments for gene expression analysis. The young spikes of Wangshuibai and its susceptible mutant NAUH117 were inoculated by *Fg* using the single-floret inoculation method [45].

### Primers designing

All primers used for RACE (Rapid Amplification of the cDNA ends, RACE), RT-PCR, qRT-PCR and plasmid constructions are listed in Table 3.

**Table 3:** Sequences of the gene-specific primer pairs used in the study.

Primers	Sequence 5' to 3'
RT-PCR Primers from wheat EST for *SGT1-1*	*SGT1-1-F*: CGCCATGTTCACTGACGA; *SGT1-1-R*: GATCCATGGTCGGAGCAA
RACE nest Primers	*5’-SGT1-S1*: AATCTCCTCAACCTCCAGCA; *3’-SGT1-A1*: TTGTTATCAGGGCCTGTTCC
RT-PCR Primers for *Ta-SGT1*	*SGT1-RT-F*: ACTGAGGCTGTAGCTGATG; *SGT1-RT-R*: CATATCTTCATCAGCATCAC
RT-PCR Primers for *Hv-SGT1*	*Hv-SGT1-RT-F*: TCGGATCTGGAGAGCAAG; *Hv-SGT1-RT-R*: TCCTGGTCCTCAGCTT
RT-PCR Primers for *Tubulin*	*Tubulin-F* AGAACACTGTTGTAAGGCTCAAC; *Tubulin-R*: GAGCTTTACTGCCTCGAACATGG
Chromosomal Location	*SGT1-F4*: AGGCTGTAGCTGATGCCA; *SGT1-R5*: TCTTCATATGCATCTGGTGACT
Sub-cellular localization Primers	*Hv-SGT1-SalⅠ-F*: CGG T C G A CATGGCCGCCGCCGCCG; *Hv-SGT1-NcoⅠ-R*: CGC C A T G G AATACTCCCACTTCTTG
VIGS Primers	*Hv-SGT1-VIGS-F*: GCTG C T A G CAGCTGATGCCAACAAAG; *Hv-SGT1-VIGS-R*: GCTG C T A G CATACGATCACACTCCT
Over-expression Primers	*Hv-SGT1-BamHI-F*: CGCG G A T C CTCGACGCAGACATGG; *Hv-SGT1-KpnI-R*: CG G T A C CTCATTAATACTCCCAC
Identification Primers for *Hv-SGT1*	*CaMV35S-F*: AGTTCATTTCATTTGGAGAGAACAC; *Hv-SGT1-R4*: CAGCTGGAACACCCTTAGC
qRT-PCR Primers for *Hv-SGT1*	*Hv-SGT1-F*: ATACTCCCACTTCTTG; *Hv-SGT1-R*: AGCTGATGCCAACAAAG
qRT-PCR Primers for *Ta-PR9* (EU264058.1)	*F*: AGGCTGTGGTTTTTGTGCTT; *R*: GCTTAAAGCTGAGGCTGCAT
qRT-PCR Primers for *Ta-NADPHOX* (AY561153.1)	*F*: ATGCTCCAGTCCCTCAACCAT; *R*: TTCTCCTTGTGGAACTCGAATTT
qRT-PCR Primers for *Ta-CAT* (HM989895.1)	*F*: TGCCTGTGTTTTTTATCCGAGA; *R*: CTGCTGATTAAGGTGTAGGTGTT
qRT-PCR Primers for *Ta-APX* (EF555121.1)	*F*: GGTTTGAGTGACCAGGACATTG; *R*: GCATCCTCATCCGCAGCAT
qRT-PCR Primers for *Ta-GST* (AJ441055)	*F*: GGAGCACAAGAGCCCCGAGC; *R*: CGGGTTGTAGGTGTGCGCGT
qRT-PCR Primers for *Ta-GPOX* (AJ010455.1)	*F*: AACTACCCGCTCTGCTCCT; *R*: GCCTTGGTCCTTGTACTTCG
qRT-PCR Primers for *Ta-NPR1* (AX049430.1)	*F*: CTGTCCGACTTTGTGAGCATA; *R*: CCCGCTGTCATTCTTCAGGTTG
qRT-PCR Primers for *Ta-PR5* (AF384146.1)	*F*: CAAGCAGTGGTATCAACGCAGAG; *R*: GTGAAGCCACAGTTGTTCTTGAT
qRT-PCR Primers for *Ta-PR2* (DQ090946)	*F*: GCGTGAAGGTGGTGATTT; *R*: GTGCCCGTTACACTTGGAT
qRT-PCR Primers for *Ta-PR3* (CK207575)	*F*: ACCTCCTTGGCGTCAGCT; *R*: TCGCACCATTATTCCCTT
qRT-PCR Primers for *Ta-OPDA* (JQ409278.1)	*F*: CCATAAACGCCATCAAAGCAGG; *R*: TGCATCGGGTTCGAGTCATAGG
qRT-PCR Primers for *Ta-ERF* (GU452719.1)	*F*: CACCTTGACCTCCTCCTCTTCGC; *R*: TTGTTCCCTTTGGACGCCAGG
qRT-PCR Primers for *Ta-PR10* (CV778999)	*F*: ACGGAGCGGATGTGGAAG; *R*: GCCACCTGCGACTTGAGC
qRT-PCR Primers for *Ta-COI1* (HM447645.1)	*F*: AAGGAGTTGCTGCTTTAGTGAACG; *R*: TCAGAGTGGGTCGCTTTTACTTG

### Isolation of the cDNA of SGT1 from *H. villosa* and the translocation line 92R137

Twenty-six wheat EST sequences with high homology to barley *SGT1* were found and aligned for the design of specific primers. First-strand cDNA was reversely transcribed using 2 µg of total RNA extracted from either the powdery mildew–resistant line 92R137 or *H* *. villosa* 24 h after inoculation. A 600-bp fragment was obtained by RT-PCR using the specific primers *SGT1*-1-F and *SGT1*-1-R (Table 3). The 3ʹ-RACE was conducted using the 3ʹ-RACE System for Rapid Amplification of cDNA Ends kit (Invitrogen, USA), and the 5ʹ-Race was conducted using the 5ʹ-Full RACE Core Set (Takara, Japan). The primers *Hv-SGT1-*5’RACE and *Hv-SGT1-*3’RACE (Table 3) were used for PCR-mediated amplification of two fragments, which were 1100 bp and 400 bp. These sequences were cloned, sequenced, and spliced in order to clone the full-length cDNA sequences of both *Ta-SGT-92R137* and *Hv-SGT1*.

### Sequence analysis and phylogenetic tree construction

The putative function of the cloned genes were analyzed using the BLAST software (http://www.ncbi.nlm.nih.gov/blast/), the ORF was predicted by the ORF Finder software (http://www.ncbi.nlm.nih.gov/gorf/gorf.html), the multiple sequence aligment was conducted by ClustalX 1.83 program, and the phylogenetic tree was constructed using the MEGA 4.0 software.

### Chromosome location of *Hv-SGT1* gene

DNA samples from the *H* *. villosa* , *T* *. durum* - *H* *. villosa* amphiploid, DA1V-DA7V, and Chinese spring were used as templates for PCR analysis to determine the chromosomal location of the *Hv-SGT1* in the *H* *. villosa* genome using the primer pairs *Hv-SGT1*–F4 and *Hv-SGT1*–R5 (Table 3). The PCR was performed in 25-µl reaction volumes including 1× PCR buffer, 2 mmol/l MgCl_2_, 0.15 mmol/l dNTPs, 20 ng of each primer, 2 µl template and 1U Taq DNA polymerase (Takara Bio, Japan). The conditions for thermal cycling involved incubation at 94°C for 3 min, followed by 33 cycles that each involved 94°C for 45 s, 50°C for 45 s, and 72°C for 1 min. The PCR products were separated in 8% non-denaturing polyacrylamide gels.

### qRT-pcr and RT-PCR for gene expression analysis

Total RNA was isolated using Trizol Reagent (Invitrogen, USA) and quantified with a NanoDropTM 1000 spectrophotometer (Thermo, Fisher Scientific, USA). The first-strand cDNA was synthesized using 2 µg of total RNA by the AMV reverse transcriptase (Takara) following the manufacturer’s instruction. For expression pattern analysis, *Hv-SGT1*-RT-F and *Hv-SGT1*-RT-R (Table 3) specific primers for *Hv-SGT1, SGT1*-RT-F and *SGT1*-RT-R (Table 3) primers for *Ta-SGT1-92R137*, were used. To evaluate the effectiveness of the gene silencing by VIGS, the expression of the *Hv-SGT1* was analyzed by qRT-PCR with a pair of primers (*Hv-SGT1*-qRT-F and *Hv-SGT1*-qRT-R, Table 3) specific to *Hv-SGT1* using the tubulin as the internal control for normalization. The PCR reaction was performed in 25 µl of reaction mixture containing 1×SYB Premix Ex Taq (Takara), 0.2 µM of each primer, 1× Rox Reference Dye, and about 30 ng cDNA per sample using the ABI Prism 7500 system (Applied Biosystems, USA). The program used was as follows: 1 min at 95°C, followed by 40 cycles at 95°C for 10 s, 60°C for 20 s, and 72°C for 40s. Three independent biological replications were performed for each treatment. Dissociation curves were generated for each reaction to ensure specific amplification. Threshold values (CT) generated from the ABI PRISM 7500 Software Tool (Applied Biosystems) were used to quantify relative gene expression using the comparative 2^–ΔΔCT^ method [46]. The expression of *Hv-SGT1* and a set of H_2_O_2_, SA and JA pathogenesis-related genes in both transgenic plants and *Hv-SGT1* silencing plants were analyzed by the qRT-PCR, using the tubulin gene (amplified with specific primers, Table 3) as the internal control. The PCR was performed in 25 µl of reaction mixture containing. The program used was as follows: 3 min at 94°C, followed by 27 cycles at 94°C for 30 s, 55-60°C for 30 s, and 72°C for 40s, and then retained at 10°C.

### Sub-cellular localization of the *Hv-SGT1* in epidermal cells of onion and *H. villosa*

*Sal* I and *Nco* I sites were added to the 5ʹ and 3ʹ ends of the full length open reading frame of *Hv-SGT1*, respectively, with the stop codon deleted by appropriate design of the *Hv-SGT1- Nco* I -R primer. The PCR product and the sGFP (s65t) vector were cut by *Sal* I and *Nco* I, and the fragments were ligated to produce the fusion gene expression vector p35S::Hv-SGT1-GFP::Nos3. After confirmation of the recombinant construct, it was delivered either to epidermal cells on the adaxial surfaces of layers peeled from an onion or to *H* *. villosa* leaves by particle bombardment, as described by Chen et al. [25]. The sub-cellular localization of the Hv-SGT1-GFP fusion protein was observed using a Leica confocal microscope (SP2) fitted with 20× numerical aperture plan apochromat objectives (the nucleus was stained with DAPI).

### Functional analysis of *Hv-SGT1* by VIGS

A pair of primers *Hv-SGT1*-VIGS-F/R with *Nhe*Ⅰ sites was introduced to the 5' and 3' end of the *Hv-SGT1* partial sequence (202bp fragment flanking the stop codon) and then was reversely inserted into the RNAγ gammab strand of the BSMV. The methods of recombinant vector RNAγ gammab: *Hv-SGT1* construction, plasmid linearization, in vitro transcription and virus infection were as described as Cao et al. [23]. The 5^th^ full expanded young leave inoculated with BSMV were detached from the plant and mounted on the culture medium in a Petri dish. Fresh powdery mildew spores were brushed off onto the leaf surface. After fourteen hours incubation in dark, the Petri dish was placed in a chamber in which the temperature was set at 25°C and the photoperiod was 14/10 h light/dark. Five days later leaves were fixed, bleached and stained with Commassie blue for observation of fungal development in bright field under Olympus BX-60 Stereo-Fluoroscope. 10 leaves per sample were observed to evaluating the effect of silencing. The 5^th^ leaves challenged with BSMV: *Hv-SGT1* or not were also used to check expression level of *Hv-SGT1* on the iCycler iQ^TM^ Multi-Color Real Time PCR Detection System (BioRad).

### Development and molecular characterization of *Hv-SGT1* transgenic wheat plants

The over expression vector pBI220-35S::Hv-SGT1::Nos3 was constructed by inserting *Hv-SGT1* into the plant expression vector pBI220, using the forward primer (*Hv-SGT1*-BamH1-F) and the reverse primer (*Hv-SGT1*-Kpnl-R) for amplifying the ORF fragment. Another plant expression vector pAHC25 containing report gene β-glucuronidase gene (*Gus*) and the herbicide tolerance gene (*Bar*) driven by the ubiqutin (*Ubi*) promoter was used as the co-transformation vector. Putative transgenic wheat plants (Yangmai 158) were produced by particle bombardment of calli cultured from immature embryo and subsequently selected by biolaphos in callus maintain, differentiation and regeneration medium as described [47]. Fully developed plants were transferred into small pots in the greenhouse [20–25°C, 14/10 h (d/night) photoperiod].

Total genomic DNA was isolated from fresh leaves of transgenic plants and untransgenic control using a modified SDS-Na^+^ salts protocol [48]. PCR amplification use primers specific for the *Hv-SGT1-R4* and the promoter *CaMV35S-F* (Table 3). PCR procedure was the same as described in chromosome location and the PCR products were separated in 0.8% agarose gel. The PCR products amplified from the PCR positive transgenic plants and Yangmai 158 were transferred to Hybond^TM^-N^+^ membrane (Amersham Biosciences, UK) and southern blotting was conducted using the partial coding region of *Hv-SGT1* and the promoter *CaMV35S* as the probe which was labeled with digoxigenin by PCR using specific primer *CaMV35S-F* and *Hv-SGT1*-R4 (Table 3). Southern blot hybridization was the same as described in the copy number of *Hv-SGT1* detecting. qRT-PCR analysis of the expression levels of *Hv-SGT1* in the transgenic lines and wild Yangmai158 was taken as the same as described in VIGS analysis.

### Tests of *Bgt* resistance in plants that over-express *Hv-SGT1*

The characterized positive transgenic T_2_ plants (151 individuals derived from four positive T_0_ lines) were identified by PCR analysis at the seedling stage, and were grown under both greenhouse and field conditions. The WT line Yangmai 158 was used as a negative control. Transgenic T_2_ plants were planted in the greenhouse, with ten T_2_ seeds derived from each T_1_ accession planted in a row. The susceptible cultivar Suimai 3 were planted in every fifth row for *Bgt* propagating and spraying. To estimate the resistance of adult T_2_ plants following natural infection with the native population of the pathogen, the mildew was assessed on the upper two leaves as a visually estimated percentage of leaves covered with mildew. The *Bgt* infection types (ITs) were scored on a 0–9 scale [49] and disease indexes (DI) of the plants were calculated as described by Chen et al. [25]. The ANOVA (analysis of variance) was performed to determine the significance of differences between the transgenic plants and wild type. The *Bgt* hyphae were stained with DioC6 and photographed using an Olympus MVX10 microscope (Japan). Wheat leaves of four transgenic lines and Yangmai158 control infected with *Bgt* were sampled at 48 hpi and stained as described [24]. Bleached leaf segments were examined with an Olympus a BX-51 microscope (Olympus, Japan) for infection sites. The the average number of hyphal branches and average hyphal length were calculated by DP-BSW software, and the final data of each index was the mean of at least 30 infection sites for each of the five randomly selected leaf segments per line. Standard deviations and a paired sample *t*-test for statistical analysis were performed with SAS software.

### Evaluation of FHB resistance in transgenic plants that over-express *Hv-SGT1*

Transgenic T_1_ (164 individuals derived from four positive T_0_ lines during 2010–2011) and T_2_ (151 individuals derived from four positive T_0_ lines during 2011–2012) were planted under greenhouse and field conditions, respectively. Yangmai 158 was used as the negative control, and Sumai 3 and Mianyang 85-45 were used as the disease-resistant and disease-susceptible controls, respectively. The resistance to wheat FHB was evaluated using single floret injection, and at least three heads per plant were inoculated. The percentages of diseased spikelets were calculated 20 days after initial inoculation. Thirty characterized positive transgenic plants were recorded for each line and the average percentages of diseased spikelets were calculated. Analysis of variance (ANOVA) was performed to determine the significance of differences between the transgenic and wild-type plants.

### Histochemical detection of H_2_O_2_ using DAB

We detected H_2_O_2_ using the DAB (Bio Basic Inc., Shanghai, China) staining method described by Thordal-Christensen et al. [50] Eight hours before sampling, inoculated primary leaves of wheat were cut, and the cut ends were placed in a solution containing 1 mg/ml DAB dissolved in NaOH-acidified (pH 3.8) distilled water. Leaves were incubated for an additional 8 h to allow DAB uptake and reaction with H_2_O_2_ and peroxidase. The inoculated wheat leaves were then cut into 1.5-cm segments at the indicated time (12 or 24 h) and discolored in boiling 95% ethanol for 10 min before being cleared in saturated chloral hydrate. The cleared leaf segments were then stored in 50% glycerol. For microscopic observation, the treated leaf segments were mounted on glass slides in 50% glycerol, and examined (200×) using an Olympus microscope (Japan). Four leaf pieces from each transgenic line and wild-type plant were observed. Each point represents at least 100 infection sites on each leaf piece. Bars represent standard deviation. Standard deviations and a paired sample t-test for statistical analysis were performed using the SAS software. The NADPH oxidase activity in wheat leaf tissues was assayed as described [51].
